# Multimodal contrastive learning for spatial gene expression prediction using histology images

**DOI:** 10.1093/bib/bbae551

**Published:** 2024-10-29

**Authors:** Wenwen Min, Zhiceng Shi, Jun Zhang, Jun Wan, Changmiao Wang

**Affiliations:** School of Information Science and Engineering, Yunnan University, East Outer Ring Road, Chenggong District, Kunming 650500, Yunnan, China; School of Information Science and Engineering, Yunnan University, East Outer Ring Road, Chenggong District, Kunming 650500, Yunnan, China; School of Information Science and Engineering, Yunnan University, East Outer Ring Road, Chenggong District, Kunming 650500, Yunnan, China; School of Information and Engineering, Zhongnan University of Economics and Law, 182 South Lake Avenue, East Lake New Technology Development Zone, Wuhan 430073, Hubei, China; Medical Big Data, Shenzhen Research Institute of Big Data, Longxiang Boulevard, Longgang District, Shenzhen 518172, Guangdong, China

**Keywords:** spatial transcriptomics, histology images, multimodal contrastive learning, transformer encoder

## Abstract

In recent years, the advent of spatial transcriptomics (ST) technology has unlocked unprecedented opportunities for delving into the complexities of gene expression patterns within intricate biological systems. Despite its transformative potential, the prohibitive cost of ST technology remains a significant barrier to its widespread adoption in large-scale studies. An alternative, more cost-effective strategy involves employing artificial intelligence to predict gene expression levels using readily accessible whole-slide images stained with Hematoxylin and Eosin (H&E). However, existing methods have yet to fully capitalize on multimodal information provided by H&E images and ST data with spatial location. In this paper, we propose mclSTExp, a multimodal contrastive learning with Transformer and Densenet-121 encoder for Spatial Transcriptomics Expression prediction. We conceptualize each spot as a “word”, integrating its intrinsic features with spatial context through the self-attention mechanism of a Transformer encoder. This integration is further enriched by incorporating image features via contrastive learning, thereby enhancing the predictive capability of our model. We conducted an extensive evaluation of highly variable genes in two breast cancer datasets and a skin squamous cell carcinoma dataset, and the results demonstrate that mclSTExp exhibits superior performance in predicting spatial gene expression. Moreover, mclSTExp has shown promise in interpreting cancer-specific overexpressed genes, elucidating immune-related genes, and identifying specialized spatial domains annotated by pathologists. Our source code is available at https://github.com/shizhiceng/mclSTExp.

## Introduction

With the rapid development of spatial transcriptomics (ST) technology, we may gain a more comprehensive understanding of gene expression patterns within complex biological systems [1]. Compared to traditional transcriptomics, this technology enables high-throughput RNA sequencing across entire tissue sections while preserving spatial information regarding cell locations within tissue slices, allowing researchers to visually observe the spatial distribution of gene expression [2, 3]. The insights afforded by this technology extend beyond gene expression, offering novel perspectives on cell–cell interactions and molecular signaling pathways within the research domain [4]. Despite these advancements, effectively harnessing the unique attributes of ST data to investigate spatial gene expression patterns and develop spatial gene detection methodologies at varying resolutions remains challenging [5]. In order to fully utilize spatial location information, several novel computational methods have been developed for spatial domain recognition (SPACEL [6], SEDR [7], STAGATE [8], CCST [9], etc.) and exploring super-resolution gene expression patterns (BayesSpace [5], iStar [10], TESLA [11], etc.).

Despite the rapid development of ST technology, the cost of generating such data remains relatively high, thus limiting the applicability of ST technology in large-scale studies. In contrast, whole-slide images (WSIs) [12–14] stained with Hematoxylin and Eosin (H&E) are more readily available, cost-effective, and widely used in clinical practice. Using H&E images to predict ST gene expression profiles has become a more common and cost-effective research approach [15, 16]. In recent studies, Schmauch et al. confirmed the feasibility of using H&E images to predict ST gene expression profiles [17]. Their developed HE2RNA method performed excellently in capturing subtle structures within H&E images, revealing critical tumor regions specific to certain cancer types.

Pathological images (such as H&E images) reveal the cellular structure, morphological features, and pathological changes within tissues, while ST technology elucidates gene expression patterns and their spatial distribution. Integrating this information is crucial for a deeper understanding of disease pathogenesis, prognosis assessment, and the development of personalized treatment strategies [18–20]. Several methods, such as STnet [21], HisToGene [15], His2ST [22], THItoGene [23], and Bleep [24], have been explored for integrating histopathological images with transcriptomic data. STnet segments tissue slice images into different patches and encodes each patch using DenseNet [25], which are then embedded into the feature space and projected onto the dimension of gene expression through fully connected layers. HisToGene employs a Vision Transformer (ViT) [26] to encode each patch and enhances spatial relationships between patches through a self-attention mechanism. His2ST introduces a graph neural network (GNN) [27] to better learn spatial relationships between spots, thus improving performance. THItoGene utilizes H&E images as input and employs dynamic convolutional and capsule networks to capture signals of potential molecular features within histological samples. Bleep utilizes a contrastive learning [28] approach, introducing image and gene expression encoders to learn joint embedding in space.

However, none of the aforementioned methods have effectively integrated the multimodal information provided by H&E images and ST data with spatial location. To address this issue, we propose mclSTExp, a multimodal deep learning approach utilizing Transformer and contrastive learning architecture. Inspired by the field of natural language processing, we regard the spots detected by ST technology as “words” and the sequences of these spots as “sentences” containing multiple “words”. We employ a self-attention mechanism to extract features from these “words” and combine them with learnable position encoding to seamlessly integrate the positional information of these “words”. Subsequently, we employ a contrastive learning framework to align the combined features with image features. Our experimental results demonstrate that mclSTExp accurately predicts gene expression in H&E images at different spatial resolutions. This is achieved by leveraging the features of each spot, its spatial information, and H&E image features. Additionally, mclSTExp demonstrates the ability to interpret specific cancer-overexpressed genes, immunologically relevant genes, preserve the original gene expression patterns, and identify specific spatial domains annotated by pathologists (Supplementary Note 1).

## Materials and methods

### Dataset description and data preprocessing

The proposed mclSTExp and competing methods are evaluated on three real datasets (Supplementary Table S1). Each dataset includes H&E images, spatial gene expression data, and spot coordinates as follows:

- The lower resolution (100${\mu }m$ per spot) **HER2+** dataset [15] contains 36 tissue sections obtained from eight patients. We selected and retained 32 sections from seven patients, ensuring that each section had a minimum of 180 spots.
- The lower resolution (100${\mu }m$ per spot) **cSCC** dataset [29] contains 12 tissue sections obtained from four patients, with each patient contributing three sections.
- The higher resolution (55${\mu }m$ per spot) **Alex+10x** dataset contains 9 breast cancer tissue samples, including 3 samples (2 fresh-frozen and 1 formalin-fixed-paraffin-embedded, FFPE) from 10x Genomics [30] and six samples obtained from Swarbrick’s laboratory [31].

For H&E images, we partitioned a $W \times H$ pixel region around each sequencing spot based on its positional coordinates, where $W$ and $H$ denote the width and height of the patches, respectively. Both $W$ and $H$ are set to 224, corresponding to the diameter of each spot in the ST data.

For the spatial gene expression data, we initially identified common genes across all tissue sections in the training ST data. Subsequently, we chose the top 1000 highly variable genes (HVG) in each tissue section, excluding genes expressed in fewer than 1000 spots across all tissue sections. The counts for each spot were normalized by dividing the total counts for that spot and then scaled by a factor of 1000 000. Finally, the values were transformed to a natural log scale, i.e. $\log (x+1)$.

After pre-processing the ST datasets, HER2+ retained 11 548 spots with 785 genes, cSCC retained 8671 spots with 171 genes, and Alex+10x retained 25 914 spots with 685 genes, as detailed in Supplementary Table S1. We paired image patches with spots, resulting in $N^{2}$ squared samples of (patches, spot) pairs. Since the patches are divided based on the positional coordinates of the spots, spots and patches with the same positional coordinates naturally form positive sample pairs. Among these, there are $N$ positive samples, representing correctly matched (patches, spot) pairs, and the remaining $N^{2}-N$ samples are negative instances, representing incorrectly matched (patches, spot) pairs.

### Overview of mclSTExp

In pathological tissues, the majority of genes exhibit low expression levels or minimal variation across most cells. These low-expression or low-variation genes can introduce noise, interfering with data analysis. Conversely, HVG are often closely associated with cellular functional states and biological processes. Selecting these genes helps to uncover significant biological differences between cells. Therefore, our goal is to predict the expression profiles of HVG in tissue sections.

**Figure 1:**
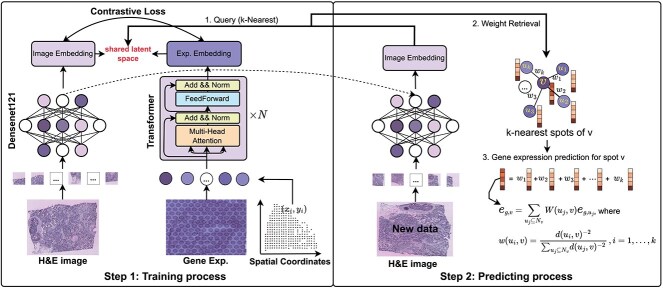
The architecture of the proposed mclSTExp model. Step 1: mclSTExp seamlessly integrates spot features with their positional information using the self-attention mechanism of Transformer. Subsequently, it fuses H&E image information through contrastive learning, thus learning a multimodal embedding space enriched with diverse features. Step 2: Projected image patches into the learned multimodal embedding space to query the expressions of the nearest k spotsl; inferred the gene expression of the test image by weighted aggregation of these queried spot expressions.

The proposed mclSTExp learns a multimodal embedding space from H&E images, spot gene expression data, and spot positional information (Fig. 1). Specifically, the image is passed through an image encoder to capture visual features, while the spot’s gene expression data along with its positional encoding are input to the Spot encoder to capture fused features incorporating spatial information. Contrastive learning is then applied to the obtained visual features and fused features, maximizing the cosine similarity of embedding for truly paired images and gene expressions, while minimizing the similarity for incorrectly paired embedding. This facilitates the fusion of image features, thereby further enhancing the model’s representational capacity.

To predict spatial gene expression from an test image, the image is fed into the image encoder to extract its visual features. Subsequently, the cosine similarity is computed between the obtained visual features and the features of $N$ spots (consistent with the training process). The top $k$ spot features with the highest similarity scores are selected, and their corresponding ground truth gene expressions are weightedly aggregated to infer the gene expression of the test images.

### Image and spot encoders

We segment 224 $\times $ 224 pixel image patches from H&E images based on the positions of spots. For each extracted image patch $\text{Patch}_{\text{i}}$, we utilized pre-train DenseNet-121 to embed it into feature $\text{z}_{\text{i}}^{\text{Patch}}$, followed by projecting it into a feature space using a projection layer. In contrast to the skip connections in ResNet [32], the dense connectivity mechanism in DenseNet-121 enhances the reusability of features, aiding the neural network in capturing image features more effectively and thereby strengthening the model’s expressive capability [25].


(1) \begin{align*} & \begin{aligned} \text{z}^\text{patch}_{i} = \text{Densenet-121}(\text{patch}_\text{i}), \end{aligned} \end{align*}

(2) \begin{align*} & \begin{aligned} \text{h}^\text{patch}_{i} = \text{MLP}\left(\text{z}_\text{i}^\text{patch}\right).\qquad\qquad\ \ \ \ \end{aligned} \end{align*}

Inspired by the field of natural language processing, we regard the spots detected by ST technology as “words” and the sequences of these spots as “sentences” containing multiple “words”. We employ a self-attention mechanism to extract features from these “words” and combine them with learnable position encoding to seamlessly integrate the positional information of these “words”. Multi-head attention is an extension of the attention mechanism, enhancing the model’s ability to capture complex patterns and global information in input sequences by simultaneously learning multiple independent sets of attention weights as follows:


(3) \begin{align*}& \begin{aligned} \text{MHSA}(Q, K, V) = [head_{1}, \ldots, head_{n}] W_{0}, \end{aligned}\end{align*}

where $W_{0}$ represents the weight matrix used for aggregating the attention heads, while $n$ denotes the number of heads. Additionally, $Q$, $K$, and $V$ correspond to Query, Key, and Value, respectively. The attention mechanism is defined as follows:


(4) \begin{align*} & \begin{aligned} \text{head}_{i} = \text{Attention}\big(QW_{i}^{Q}, KW_{i}^{K}, VW_{i}^{V}\big), \end{aligned} \end{align*}

(5) \begin{align*} & \begin{aligned} \text{Attention}(Q, K, V) = \text{softmax}\left(\frac{QK^{T}}{\sqrt{d_{k}}}\right)V, \end{aligned} \end{align*}

where $W_{i}^{Q}$, $W_{i}^{K}$, and $W_{i}^{V}$ are weight matrices. The term $\Big(\frac{\text{QK}^{\text{T}}}{\sqrt{\text{d}_{\text{k}}}}\Big)$ is called Attention Map, whose shape is $N\times N$. The term $V$ is the value of the self-attention mechanism, where $V = Q = K$.

Regarding the positional information of spots, each spot’s coordinates $(x, y)$ are represented by a matrix of size $N\times 2$. The $x$-coordinate information is transformed into a one-hot encoding matrix $P_{x}$ of size $N\times n$, where $n$ is the maximum number of $x$-coordinates across all tissue sections. For the all datasets, $n=65536$. Then, the matrix is linearly transformed using the learnable linear layer $W_{x}$ to obtain an $N\times hvg{\_ }num$ matrix $S_{x}$ that maintains the same dimensions as the Spots ($\text{Spots}\in \text{R}^{\text{spot_num,hvg_num}}$). Similarly, the $y$-coordinate vector undergoes a similar transformation to obtain an $N\times hvg{\_ }num$ encoding matrix $S_{y}$. Finally, the spot feature, $x$-coordinate encoding matrix $S_{x}$, and $y$-coordinate encoding matrix $S_{y}$ are combined and passed through a multi-head attention mechanism using Equation (3):


(6) \begin{align*}& \begin{aligned} \text{z}^\text{spot}_{i} = \text{MHSA}(\text{Spot}_{i}+ \text{S}_{x}+ \text{S}_{y}), \end{aligned}\end{align*}

Then, project it into a feature space using a projection layer:


(7) \begin{align*}& \begin{aligned} \text{h}^\text{spot}_{i} = \text{MLP}\left(\text{z}_\text{i}^\text{spot}\right). \end{aligned}\end{align*}

In this feature space, the dimensions of $\text{h}^\text{patch}_{i}$ and $\text{h}^\text{spot}_{i}$ are both $N\times 256$.

We utilize a self-attention mechanism to integrate the gene expression features and spatial location features of spots. This multimodal feature representation not only integrates critical information from gene expression but also takes into account the specific spatial location of each point within the tissue image. As a result, each spot in the feature space exhibits a more distinct and enriched expression. Specifically, the partitioning of H&E image patches is based on the positions of spots. Therefore, spots and patches located at the same position inherently form a positive sample pair, while those at different positions constitute negative sample pairs.

### Contrastive learning module

We adopt a contrastive learning approach to reduce the distance between positive sample pairs and increase the distance between negative sample pairs, thereby achieving the fusion of image information. Specifically, in each batch comprising $N$ pairs of (patch, spot). We utilize the mclSTExp algorithm to simultaneously train both the image encoder and Spot encoder, aiming to construct a multimodal embedding space. The optimization objective of this space is to maximize the cosine similarity of $N$ positive sample pairs and simultaneously minimize the cosine similarity of $N^{2}-N$ negative sample pairs. We employ the loss function of CLIP [33] and fine-tune it to suit our task.

For integrating positive sample pairs, we employ a label matrix where diagonal elements represent positive sample pairs (labeled as 1), and non-diagonal elements represent negative sample pairs (labeled as 0). Subsequently, we utilize the cross-entropy loss function to achieve effective classification.

To show the overall loss of our model, we first define the cosine similarity function cos_sim between patch and spot embedding as follows:


(8) \begin{align*}& \begin{aligned} (\mathbf{h}^{\text{patch}},\mathbf{h}^{\text{spot}})=\mathbf{h}^{\text{patch}} \cdot (\mathbf{h}^{\text{spot}})^{T}, \end{aligned}\end{align*}

where the “label” matrix is defined as:


(9) \begin{align*}& \text{label} = \left(\begin{array}{cccc} 1 & 0 & 0 & \dots \\ 0 & 1 & 0 & \dots \\ \vdots & \vdots & \ddots & \vdots \\ 0 & 0 & \dots & 1 \end{array}\right)\end{align*}

And the cosine similarity function between spot and patch embedding is defined as:


(10) \begin{align*}& \begin{aligned} (\mathbf{h}^{\text{spot}},\mathbf{h}^{\text{patch}})=\mathbf{h}^{\text{spot}} \cdot (\mathbf{h}^{\text{patch}})^{T}. \end{aligned}\end{align*}

Two individual loss components, $\text{loss}_{\text{image}}$ and $\text{loss}_{\text{spot}}$, are computed using the cross-entropy loss function (ce:Loss).


(11) \begin{align*} & \begin{aligned} \text{Loss}_{\text{image}} &= \text{CE\_Loss}((\mathbf{h}^{\text{patch}}, \mathbf{h}^{\text{spot}}),\text{label}), \end{aligned} \end{align*}

(12) \begin{align*} & \begin{aligned} \text{Loss}_{\text{spot}} &= \text{CE\_Loss}((\mathbf{h}^{\text{spot}}, \mathbf{h}^{\text{patch}}),\text{label}), \end{aligned} \end{align*}

where $\text{Loss}_{\text{image}}$ is based on the similarity between the image embedding and the transpose of spot embedding, while $\text{Loss}_{\text{spot}}$ is based on the similarity between spot embedding and the transpose of image embedding.

Finally, the overall loss of our model is calculated:


(13) \begin{align*}& \begin{aligned} \text{Loss} &= \lambda \times \text{Loss}_{\text{image}} + (1 - \lambda) \times \text{Loss}_{\text{spot}}. \end{aligned}\end{align*}

where $\lambda $ is a hyperparameter that control the contribution in the loss function. Our experimental results (Supplementary Note 2, Table S8) show that setting $\lambda $ to 0.5 can improve the performance of mclSTExp. Consequently, the subsequent results are based on $\lambda $ = 0.5.

### Weight aggregation module

As illustrated in Step 2 of Fig. 1, the procedure commences by segmenting the H&E image into $N$ small patches, which are later encoded by the pre-trained image encoder. Once the image patches are represented in the joint embedding space, the process transitions to predicting the gene expression of an image. To initiate this prediction, the test image data (new data) are input into an image encoder, extracting its visual features. Within the established shared embedding space, the cosine similarity is then computed between the visual features of the test image (new data) and all spot features, maintaining consistency with the training process. Subsequently, the top $k$ spot features with the highest similarity scores are discerned. The Euclidean distance is calculated within the shared embedding space between the visual features of the image and spot features as follows:


(14) \begin{align*}& \begin{aligned} d(u,v)=\sqrt{ {\textstyle \sum_{i=1}^{n}} (u_{i}-v_{i})^{2}}, \end{aligned}\end{align*}

where $n$ represents the dimensionality of the feature space, the next step involves inferring the gene expression value for spot $v$ by using the weighted distance [11] of the gene expressions of the top $k$ spots based on their similarity scores. The weights are defined as follows:


(15) \begin{align*} & \begin{aligned} W(u_{i},v)=\frac{d(u_{i},v)^{-2}}{ {\textstyle \sum_{u_{j}\in N_{v}}d(u_{j},v)^{-2}}}, \end{aligned} \end{align*}

(16) \begin{align*} & \begin{aligned} e_{g,v}= {\textstyle \sum_{u_{j}\in N_{v}}W(u_{j},v)e_{g,u_{j}}}. \end{aligned} \end{align*}

where $e_{g,u_{j}}$ represents the observed gene expression for spot $u_{j}$.

### Baselines

In this study, we selected five representative state-of-the-art methods:

- **STnet [21]** utilized DenseNet-121 as the image encoder to extract H&E image features, which were then embedded into the feature space and projected onto the dimension of gene expression through fully connected layers.
- **HisToGene [15]** adopted a ViT as the image encoder, leveraging self-attention mechanism to extract global features, which were subsequently projected onto the dimension of gene expression through fully connected layers.
- **His2ST [22]** employed the Convmixer module to capture the internal relationships of 2D visual features within H&E images through convolution operations. Additionally, the Transformer module captured global spatial dependencies using a self-attention mechanism, while the GNN module explicitly captured the neighborhood relationships between spots.
- **THItoGene [23]** used H&E images as input and employed dynamic convolutional and capsule networks to capture signals of potential molecular features within histological samples.
- **BLEEP [24]** utilized a contrastive learning approach, introducing image and gene expression encoders to jointly learn embeddings in feature space for inferring gene expression.

### Evaluation citeria

We use PCC, Mean Squared Error (MSE), and Mean Absolute Error (MAE) to evaluate the proposed method against baselines.


(17) \begin{align*}& \begin{aligned} \text{PCC}=\frac{\text{Cov}(\text{X}_{\text{observed}},\ \text{X}_{\text{pred}})}{\text{Var}(\text{X}_{\text{observed}}) \times \text{Var}(\text{X}_{\text{pred}})}, \end{aligned}\end{align*}

where $\text{Cov()}$ is the covariance, and $\text{Var()}$ is the variance. $\text{X}_{\text{observed}}$ and $\text{X}_{\text{pred}}$ are the observed and predicted gene expression, respectively.


(18) \begin{align*} & \begin{aligned} \text{MSE}=\frac{1}{N} \sum_{i=1}^{N} (\text{X}_{\text{observed}}-\text{X}_{\text{pred}})^{2}, \end{aligned} \end{align*}

(19) \begin{align*} & \begin{aligned} \text{MAE}=\frac{1}{N} \sum_{i=1}^{N} \left | \text{X}_{\text{observed}}-\text{X}_{\text{pred}}\right |, \end{aligned} \end{align*}

PCC measures the mean correlation for each gene type, considering predictions and ground truth across all slide images. Meanwhile, MSE and MAE measure the sample deviation between predictions and ground truth for each gene type in each slide image. For PCC, a higher value indicates better performance. Conversely, for MSE and MAE, lower values indicate better performance.

**Table 1:** For the HER2+, cSCC, and Alex+10x datasets, the mean Pearson correlation coefficients (PCCs) for the predicted expression levels of ACG and the top 50 most HEG, as well as the average Mean Squared Error (MSE) and Mean Absolute Error (MAE), were calculate compared to the ground truth expressions

Methods	HER2+			
	PCC (ACG)	PCC (HEG)	MSE	MAE
STnet [21]	0.0561 $\pm $ 0.017	0.0134 $\pm $ 0.013	0.5312 $\pm $ 0.008	0.6306 $\pm $ 0.011
HisToGene [15]	0.0842 $\pm $ 0.015	0.0711 $\pm $ 0.014	0.5202 $\pm $ 0.014	0.6422 $\pm $ 0.005
His2ST [22]	0.1443 $\pm $ 0.013	0.1849 $\pm $ 0.015	0.5135 $\pm $ 0.009	0.6087 $\pm $ 0.013
THItoGene [23]	0.1726 $\pm $ 0.018	0.2809 $\pm $ 0.013	0.5012 $\pm $ 0.011	0.5956 $\pm $ 0.009
BLEEP [24]	0.1873 $\pm $ 0.005	0.2909 $\pm $ 0.016	0.6015 $\pm $ 0.016	0.5824 $\pm $ 0.004
**mclSTExp (ours)**	**0.2304 $\pm $ 0.011**	**0.3866 $\pm $ 0.021**	0.5897 $\pm $ 0.013	**0.5813 $\pm $ 0.008**
**Methods**	**cSCC**			
	**PCC (ACG)**	**PCC (HEG)**	**MSE**	**MAE**
STnet [21]	0.0012 $\pm $ 0.022	0.0018 $\pm $ 0.015	0.6806 $\pm $ 0.006	0.6404 $\pm $ 0.003
HisToGene [15]	0.0771 $\pm $ 0.024	0.0919 $\pm $ 0.012	0.6805 $\pm $ 0.012	0.6234 $\pm $ 0.007
His2ST [22]	0.1838 $\pm $ 0.011	0.2175 $\pm $ 0.016	0.6748 $\pm $ 0.017	0.6107 $\pm $ 0.006
THItoGene [23]	0.2373 $\pm $ 0.009	0.2719 $\pm $ 0.012	0.6546 $\pm $ 0.006	0.6012 $\pm $ 0.019
BLEEP [24]	0.2449 $\pm $ 0.017	0.3122 $\pm $ 0.027	0.5163 $\pm $ 0.007	0.5399 $\pm $ 0.015
**mclSTExp (ours)**	**0.3235 $\pm $ 0.019**	**0.4261 $\pm $ 0.016**	**0.4302 $\pm $ 0.005**	**0.5208 $\pm $ 0.009**
**Methods**	**Alex+10x**			
	**PCC (ACG)**	**PCC (HEG)**	**MSE**	**MAE**
STnet [21]	0.0009 $\pm $ 0.013	0.0452 $\pm $ 0.007	0.4721 $\pm $ 0.011	0.5042 $\pm $ 0.015
HisToGene [15]	0.0618 $\pm $ 0.008	0.0984 $\pm $ 0.015	0.4565 $\pm $ 0.014	0.4973 $\pm $ 0.009
His2ST [22]	0.1299 $\pm $ 0.012	0.1784 $\pm $ 0.005	0.3788 $\pm $ 0.008	0.4492 $\pm $ 0.012
THItoGene [23]	0.1384 $\pm $ 0.014	0.2156 $\pm $ 0.013	0.3672 $\pm $ 0.009	0.4315 $\pm $ 0.006
BLEEP [24]	0.1552 $\pm $ 0.009	0.2825 $\pm $ 0.012	0.2593 $\pm $ 0.013	0.4050 $\pm $ 0.015
**mclSTExp (ours)**	**0.1949 $\pm $ 0.011**	**0.3611 $\pm $ 0.018**	**0.2329 $\pm $ 0.006**	**0.3897 $\pm $ 0.011**

In the assessment of spatial clustering performance, we employ the Adjusted Rand Index (ARI) to measure the correlation between the clustering outcomes and the actual pathological annotation regions. The ARI can be mathematically expressed as follows:


(20) \begin{align*}& \text{ARI}= \frac{ {\textstyle \sum_{ij}\binom{n_{ij}}{2}}-\frac{\left [ {\textstyle \sum_{i}\binom{a_{i}}{2}\sum_{j}\binom{b_{j}}{2}}\right]} {\binom{n}{2}}}{\frac{1}{2} \left [\sum_{i}\binom{a_{i}}{2}+\sum_{j}\binom{b_{j}}{2}\right ]- \frac{\left [ {\textstyle \sum_{i}\binom{a_{i}}{2}\sum_{j}\binom{b_{j}}{2}}\right]} {\binom{n}{2}}},\end{align*}

where $a_{i}$ and $b_{j}$ are the number of samples appearing in the $i-th$ predicted cluster and the $j-th$ true cluster, respectively. $n_{ij}$ means the number of overlaps between the $i-th$ predicted cluster and the $j-th$ true cluster. The ARI is a metric with a scale ranging from -1 to 1. A value nearing 1 signifies a stronger alignment between the clustering results and the true labels.

Additionally, the Normalized Mutual Information (NMI) is another measure utilized for evaluating clustering performance, defined as:


(21) \begin{align*}& \text{NMI} = \frac{{I(X;Y)}}{{\sqrt{H(X) \cdot H(Y)}}}.\end{align*}

where $I(X;Y)$ denotes the mutual information between the predicted clustering $X$ and the true clustering $Y$, and $H(X)$ and $H(Y)$ represent the entropies of $X$ and $Y$, respectively.

## Results

### Experiment settings

We employed a grid search strategy to systematically explore the combinations of hyperparameters. Each combination was evaluated based on performance metrics on the validation set. For instance, we tested a range of learning rates (1e-5, 1e-4, 1e-3, 1e-2) to identify the optimal value that ensures stable convergence. Additionally, we experimented with various embedding dimensions (128, 256, 512, 1024) to determine the dimensionality that captures the most relevant features while maintaining computational efficiency. Regularization was performed using different weight decay values (0.0001, 0.001, 0.01, 0.1) to prevent overfitting.

mclSTExp is trained from scratch for 90 epochs on the HER2+, cSCC datasets, and 15 epochs on the Alex+10x dataset. A batch size of 128 was used during training. The learning rate was set to $1 \times 10^{-4}$, and the weight decay was $1 \times 10^{-3}$. All experiments are conducted using NVIDIA RTX 4090 GPUs with the AdamW optimizer.

In mclSTExp, a two-layer Transformer is employed as the spot encoder, with 8 attention heads, each with a dimensionality of 64. Additionally, within the contrastive learning module, the temperature hyperparameter was set to 1, and the dimensionality of the multimodal embedding space was specified as 256.

To evaluate the predictive accuracy of gene expression data, we employed a leave-one-out cross-validation training approach, where one tissue slice was held out as the test set, and the remaining slices were utilized as the training set. Leave-one-out cross-validation is a specific cross-validation technique used to evaluate the performance of machine learning models. Specifically, each time, all data except one sample are used for training, and the single remaining sample is used for validation. This process is repeated until every sample has been used once as a validation set. This approach ensures that each sample is systematically used as a validation set, providing a comprehensive and accurate assessment of the model’s performance.

**Figure 2:**
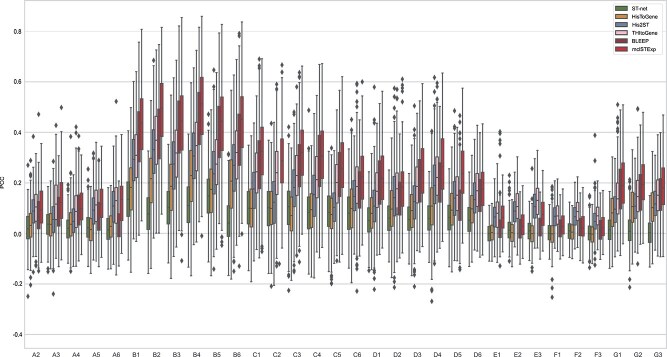
Evaluation of gene expression prediction on the HER2+ datasets by the PCC (ACGs) between the observed and predicted gene expression by STnet [21], HisToGene [15], His2ST [22], THItoGene [23], BLEEP [24], and mclSTExp.

### mclSTExp can improve the prediction accuracy

To assess the performance of mclSTExp, we analyze the HER2+ breast cancer dataset, which includes 32 tissue sections, the cSCC dataset with 12 tissue sections, and the Alex+10x dataset with 9 slices (Table 1). For the evaluation of gene expression prediction accuracy, we conduct leave-one-out cross-validation. Specifically, for each dataset, we used one slice as the test set and the remaining slices as the training set. For each tissue section, we computed the PCC for all considered genes (ACG) as well as the top 50 highly expressed genes (HEG), along with the MSE and MAE for ACG. Subsequently, the average values of PCC (ACG), PCC (HEG), MSE, and MAE across all tissue sections were calculated to evaluate the overall model performance. We compared mclSTExp with five other recently developed advanced methods for predicting spatial gene expression. Considering that the gene expression prediction task emphasizes capturing relative changes, we prefered evaluation metrics related to PCC. As shown in Table 1, mclSTExp achieved the highest average PCC for both ACG and HEG across these three datasets. Specifically, the PCC (ACG) of mclSTExp was 23.01%, 32.09%, and 25.57% higher than that of the second-ranked method BLEEP on these three datasets, while the PCC (HEG) was 32.89%, 36.48%, and 27.82% higher, respectively. Additionally, we conducted 5-fold cross-validation experiments on the HER2+ and cSCC datasets to further validate the effectiveness of the mclSTExp method. The results (Supplementary Table S2) showed that the performance metrics obtained from 5-fold cross-validation were very similar to those from LOOCV, further demonstrating the robustness of our model’s predictive ability across different validation strategies.

**Figure 3:**
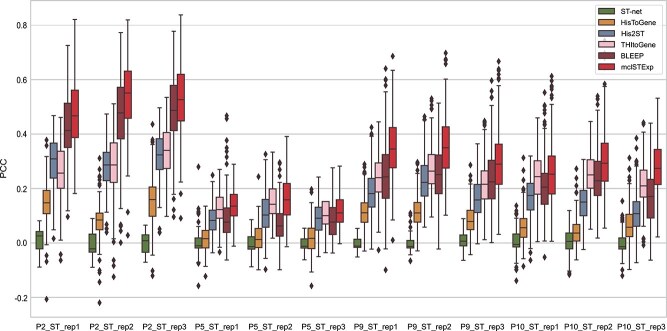
Evaluation of gene expression prediction on the cSCC datasets by the PCC (ACGs) between the observed and predicted gene expression by STnet [21], HisToGene [15], His2ST [22], THItoGene [23], BLEEP [24], and mclSTExp.

**Figure 4:**
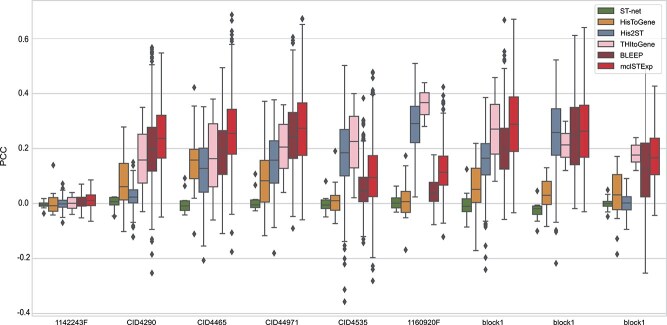
Evaluation of gene expression prediction on the Alex+10x datasets by the PCC (ACGs) between the observed and predicted gene expression by STnet [21], HisToGene [15], His2ST [22], THItoGene [23], BLEEP [24], and mclSTExp.

To examine the results of each slice individually, we visualized the PCC between the gene expression predicted by mclSTExp and the observed gene expression on each slice. As depicted in Fig. 2, mclSTExp attained the highest PCC among the 32 slices in the HER2+ dataset, achieving this distinction on 26 slices. However, for slices E1-F3, the PCC values across all methods were relatively low, suggesting potential issues with gene detection sensitivity or specificity in ST technology. Notably, mclSTExp consistently outperformed the second-ranked method Bleep across all slices. Additionally, mclSTExp demonstrated the highest PCC across all tissue sections in the cSCC dataset, as depicted in Fig. 3. Noteworthy is its substantial improvement in predicting gene expression correlation, particularly for the P10_ST_rep3 section. In Fig. 4, mclSTExp exhibited the highest PCC on 7 out of 9 slices in the Alex+10x dataset. Particularly for slices CID4535 and 1160920F, the performance of mclSTExp was lower than that of THItoGene. This result may be attributed to complex regulatory mechanisms, tumor microenvironment characteristics [30], or weak correlations between the expression of specific genes and morphological features, which posed challenges for our method in predicting their expression.

### Visualization of the predicted gene expression

**Figure 5:**
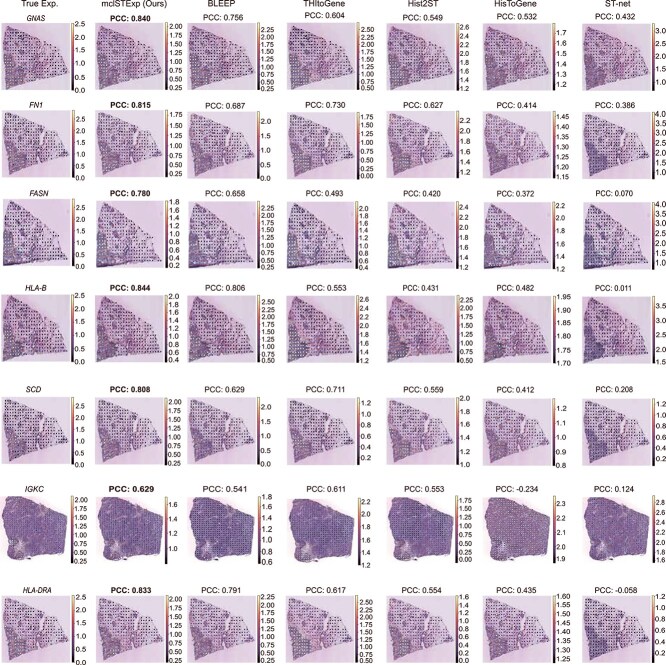
Visualize the top seven predicted genes in the HER2+ dataset based on the highest average $-\log _{10}$ (*P*-values) calculated across all tissue sections. The *P*-values are determined based on the correlation between predicted and observed gene expressions. For each of these seven genes, select the tissue section predicted by our model with the smallest *P*-value for visualization.

To further evaluate the predicted gene expression, we explored whether the gene expression predicted by mclSTExp accurately reflected the actual status of tumor-related genes. Across all datasets, we analyzed the correlation between observed gene expression and predicted gene expression, calculating correlation coefficients and *P*-values for each spot. Subsequently, we computed the average $-\log _{10}{(\text{P-values})}$ for all genes. These genes were ranked in descending order of their $-\log _{10}{(\text{P-values})}$, as detailed in Supplementary Table S3. For the HER2+ dataset, we visualized the top seven genes: *GANS*, *FN1*, *FASN*, *HLA-B*, *SCD*, *IGKC*, and *HLA-DRA*. As shown in Fig. 5, the PCCs for these genes using mclSTExp were 0.840, 0.815, 0.780, 0.844, 0.808, 0.629, and 0.833, respectively, surpassing those predicted by the second-ranked method, Bleep, by 11.1, 18.6, 18.5, 4.7, 28.4, 16.2, and 5.3%. Particularly, for the gene *IGKC*, the correlation coefficient with HisToGene was -0.234, and for the gene *HLA-DRA*, it was -0.058 with STnet.

It is noteworthy that all of the top seven genes identified by mclSTExp are closely linked to breast cancer, playing pivotal roles in its onset and progression. Elevated expression of *GANS* can activate the *PI3K/AKT/Snail1/E-cadherin* pathway, thereby facilitating the proliferation, migration, and invasion of breast cancer cells [34]. *FN1* is recognized as a potential therapeutic target or clinical prognostic marker for breast cancer, as its heightened expression is closely associated with the metastasis and deterioration processes in breast cancer [35]. *FASN* exhibits high expression in cancer stem cells, and its inhibition effectively suppresses the proliferation and survival of breast cancer cells [36]. Moreover, the proliferation, survival, and aggressiveness of breast cancer cells are closely linked to *SCD*, underscoring its potential as a therapeutic target in breast cancer treatment strategies [37]. Additionally, *IGKC* serves as a prognostic marker with significant value in predicting disease progression and survival outcomes in breast cancer patients [38].

Notably, among all the compared methods, mclSTExp showed the best performance in predicting the *HLA-B* and *HLA-DRA* genes, achieving the highest PCC scores (Fig. 5). For one thing, Human leukocyte antigen B (*HLA-B*) belongs to the major histocompatibility complex (MHC) class I molecules, primarily responsible for the presentation of intracellular peptides. A study [39] have indicated that the expression of *HLA-B* is associated with the survival and recurrence rates of breast cancer patients. For another, *HLA-DRA* is a class II MHC molecule typically expressed in professional antigen-presenting cells. Research [40] has demonstrated that *HLA-DRA* serves as a significant prognostic factor for breast cancer. Its expression levels may represent a pathway to enhance the treatment of advanced breast cancer and improve overall survival rates. Another study [41] has highlighted how cancer cells exploit various immune system functions to promote their growth. In summary, the mclSTExp method not only elucidates cancer-specific overexpressed genes but also identifies immune-related genes, providing valuable insights for cancer therapy (Table 2 and Supplementary Fig. S1).

**Table 2:** The top 50 predicted genes by mclSTExp, BLEEP, THItoGene, His2ST, HisToGene, and STNet were ranked based on the highest values of mean -log10(*P*-value) across all tissue sections in the HER2+ dataset, where the *P*-value for each tissue section was computed according to the Pearson correlation test between the predicted and observed gene expression. Genes marked in red are immune-related genes

**Rank**	**mclSTExp**	**BLEEP**	**THItoGene**	**His2ST**	**HisToGene**	**STnet**
1	GNAS	GNAS	FN1	FN1	GNAS	GNAS
2	FN1	FN1	SCD	GNAS	MYL12B	SCD
3	FASN	FASN	$\color{red}{IGKC} $	SCD	FASN	MYL12B
4	$\color{red}{HLA-B} $	SCD	FASN	MYL12B	CLDN4	STMN1
5	SCD	SPARC	GNAS	FASN	SCD	SRSF1
6	$\color{red}{IGKC} $	CCT4	$\color{red}{IGHA1} $	STMN1	FN1	HMGB2
7	$\color{red}{HLA-DRA} $	$\color{red}{IGKC} $	MYL12B	$\color{red}{IGKC} $	$\color{red}{RHOB} $	$\color{red}{RHOB} $
8	$\color{red}{CD74} $	MYL12B	CLDN4	$\color{red}{RHOB} $	TMEM123	CCT4
9	CLDN4	CLDN4	STMN1	CCT4	CCT4	TMEM123
10	UBA52	$\color{red}{CD74} $	CRABP2	TMEM123	STMN1	$\color{red}{SRGN} $
11	HSPB1	HSPB1	TMEM123	NDUFB2	NDUFB2	NDUFB2
12	MYL12B	UBA52	$\color{red}{RHOB} $	CLDN4	TXNDC17	FASN
13	STMN1	TMEM123	MUCL1	TXNDC17	SRSF1	CLDN4
14	$\color{red}{IGLC3} $	$\color{red}{VIM} $	CCT4	$\color{red}{HMGB2} $	ITGB6	ITGB6
15	$\color{red}{IGHA1} $	STMN1	TXNDC17	SRSF1	HMGB2	GPRC5A
16	$\color{red}{IGLC2} $	COL3A1	ITGB6	GPRC5A	CRACR2B	MID1IP1
17	$\color{red}{RHOB} $	$\color{red}{RHOB} $	$\color{red}{IGHG3} $	ITGB6	MID1IP1	PDCD5
18	$\color{red}{IGHG3} $	TMBIM6	KRT8	KRT8	NDRG1	TMBIM6
19	$\color{red}{VIM} $	$\color{red}{IGLC3} $	$\color{red}{IGLC2} $	TMEM14B	PRKCSH	TXNDC17
20	TMEM123	CLDN3	$\color{red}{HMGB2} $	TMBIM6	HNRNPUL2	HNRNPUL2
21	SPARC	$\color{red}{HLA-B} $	GPRC5A	$\color{red}{HLA-DRA} $	SRSF5	FN1
22	CLDN3	$\color{red}{HLA-DRA} $	SRSF1	NDUFA1	PDCD5	SRSF5
23	COL3A1	NDUFB9	NDUFB2	NDUFB3	CPNE1	TMEM14B
24	CRABP2	$\color{red}{IGLC2} $	NDRG1	CRABP2	ARF6	$\color{red}{IGHA1} $
25	NDRG1	PRKCSH	$\color{red}{HLA-DRA} $	MRPL51	TMBIM6	UBAP2L
26	TMBIM6	$\color{red}{HLA-DPA1} $	$\color{red}{C3} $	LUM	TMEM14B	CXCL10
27	CCT4	TXNDC17	NDUFB3	COL3A1	ATP5O	NDUFC1
28	$\color{red}{C3} $	BSG	TMBIM6	PDCD5	NDUFA1	ATP5H
29	MUC1	LUM	AGR2	TANC2	UBAP2L	ARF6
30	MUCL1	POSTN	NDUFA1	NDRG1	NDUFB3	NDUFB3
31	PRKCSH	FADS2	FADS2	ATP5O	$\color{red}{SRGN} $	LUC7L3
32	BSG	NDUFB2	$\color{red}{IGHM} $	$\color{red}{IGHA1} $	MRPL51	MAGEF1
33	NDUFB9	$\color{red}{IGHG3} $	HSPB1	MID1IP1	TNC	GATA3
34	NDUFB2	$\color{red}{TIMP1} $	TMEM14B	POSTN	FAM193B	MARS
35	KRT8	FLNA	PDCD5	ARF6	ATP6AP1	TEX2
36	FLNA	ATP6AP1	CRACR2B	$\color{red}{SRGN} $	FADS2	NDUFA12
37	GPRC5A	AES	MGP	SPARC	C6orf48	TNC
38	FADS2	SRSF1	LUM	TEX2	SRRT	KMT2B
39	LUM	KRT8	GATA3	FADS2	RNASET2	FARP1
40	$\color{red}{HMGB2} $	HMGB2	$\color{red}{IGLC3} $	CPNE1	$\color{red}{HLA-DRA} $	INTS8
41	$\color{red}{TIMP1} $	EIF3B	HLA-B	NDUFA12	$\color{red}{IGKC} $	CRACR2B
42	AES	NDRG1	TNC	VIM	XPO6	XPO6
43	CRACR2B	HNRNPUL2	MID1IP1	ATP5H	TANC2	AGR2
44	POSTN	$\color{red}{IGHA1} $	ATP5O	CRACR2B	MAGEF1	SRRT
45	ITGB6	$\color{red}{C3} $	MRPL51	COX5A	MRPL21	NDUFS1
46	$\color{red}{HLA-DPA1} $	CRABP2	PRKCSH	NDUFC1	DNAJC1	HLA-DPB1
47	$\color{red}{IGHM} $	IFI27	COL3A1	HSPB1	INTS8	IGKC
48	ATP6AP1	MUC1	$\color{red}{CD74} $	UBAP2L	SNRPD3	TANC2
49	TXNDC17	SRSF5	TANC2	TNC	ATP5H	NDUFA1
50	$\color{red}{S100A14} $	SAT1	HNRNPUL2	FOXP4	CLDN3	C6orf48

To assess the robustness of our method, we visualized the top seven genes using the same strategy in both the cSCC dataset and the Alex 10x dataset, as detailed in Supplementary Fig. S2 and Table S4. These genes have been previously found to be highly associated with human cutaneous squamous cell carcinoma and breast cancer in prior studies [42, 43].

Additionally, we computed the correlation matrix using the expression data of actual genes. Subsequently, hierarchical clustering was performed on this correlation matrix to obtain the clustering order of the samples. Next, we calculated the gene-gene correlations using predicted expression values obtained from various methods, reordered the correlation matrix according to the clustering order, and generated a heatmap of the correlations (Supplementary Fig. S3). The results indicate that mclSTExp effectively preserves the patterns of gene-gene co-expression and biological heterogeneity.

### Spatial region detection

**Figure 6:**
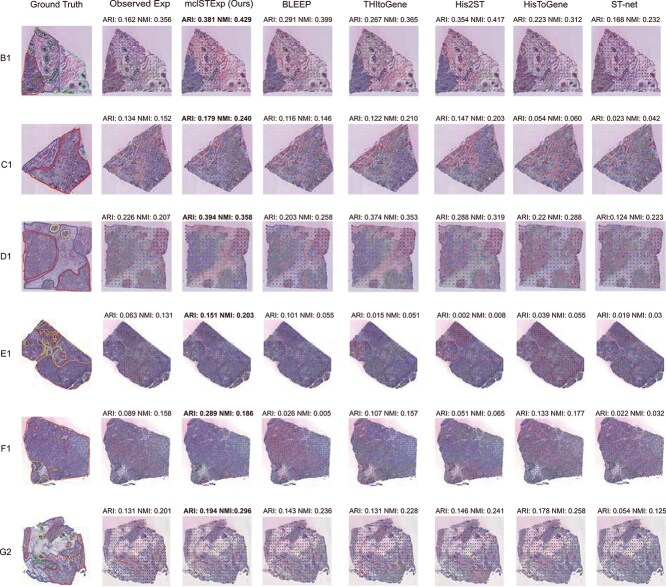
We conducted spatial clustering analysis using six H&E images annotated by pathologists from the HER2+ dataset, while “Observed Exp” represents clustering directly using the sequenced gene expression.

To evaluate the performance of various methods in identifying specific spatial domains on entire H&E images, we compared six tissue slices from the HER2+ dataset. These slices have been annotated by pathologists for spatial transcriptomic analysis. Initially, we employed PCA dimensionality reduction on the predicted data from mclSTExp, followed by Kmeans clustering. Compared to other methods, mclSTExp demonstrates the ability to accurately identify spatial domains predefined by pathologists, resulting in significant improvements in effectiveness. As shown in Fig. 6, our method achieved the highest ARI and NMI scores across all slices. Specifically, mclSTExp (avg $ARI=0.2646$, avg $NMI=0.2853$) outperforms the second-ranked method, His2ST (avg $ARI=0.1647$, avg $NMI=0.2088$), by 60.7% in terms of average ARI and by 36.6% in terms of average NMI. For the B1 slice, mclSTExp ($ARI=0.381$, $NMI=0.429$) achieves similar ARI and NMI scores as His2ST ($ARI=0.354$, $NMI=0.417$). However, for the E1 and F1 slices, mclSTExp accurately identifies their spatial structures, whereas all other methods perform poorly.

Compared to existing methods, mclSTExp treats each spot as a “word” and spot sequences as “sentences”, integrating the features and positional information of each spot through a self-attention mechanism. Additionally, by incorporating H&E image information through contrastive learning, mclSTExp learns rich representations, enabling it to sensitively capture subtle differences in H&E images, as well as the correlation between H&E images and gene expression, along with abundant spatial information. Consequently, gene expression data predicted by mclSTExp demonstrate superior performance in identifying spatial domains and better reflect the true spatial structure and biological characteristics of tissues.

## Ablation studies

To further investigate the contributions of each component of mclSTExp, we conducted a series of ablation experiments on the HER2+, cSCC, and Alex+10x datasets (Supplementary Note 2, Fig. S4, Tables S5, S6, S7 and S8).

## Discussion and Conclusion

In this study, we propose mclSTExp, a multimodal deep learning approach utilizing Transformer and contrastive learning framework for predicting gene expression from H&E images (Fig. 1). Inspired by the field of natural language processing, we regard the spots detected by ST technology as “words” and the sequences of these spots as “sentences” containing multiple “words”. We employ a self-attention mechanism to extract features from these “words” and combine them with learnable position encoding to seamlessly integrate the positional information of these “words”. Subsequently, we adopt a contrastive learning approach, maximizing the cosine similarity of positive samples to bring the correctly matched image blocks and “words” pair samples closer, while minimizing the cosine similarity of negative samples to push away incorrectly matched samples, thereby integrating image information. Through this approach, we learn a multimodal embedding space. Finally, we select the features of the top $k$ “words” with the highest cosine similarity, and then aggregate their true expression spectra by weight to infer the gene expression of the test data.

mclSTExp enables us to predict gene expression from H&E images more accurately. Based on our experimental results, the PCC (ACG) of mclSTExp was 23.01, 32.09, and 25.56% higher than that of the second-ranked method BLEEP on the HER2+ dataset, cSCC dataset, and Alex+10x dataset, respectively. Similarly, the PCC (HEG) was 32.89, 36.48, and 27.82% higher, respectively. Additionally, mclSTExp exhibits the capability to interpret cancer-specific overexpressed genes and identify specific spatial domains annotated by pathologists.

There are advanced ST data analysis frameworks such as SPACEL [6] and others. They are powerful (i) predicting spatial distributions of cell types in ST data, (ii) recognizing spatial domains across multiple slices, and (iii) construct three dimensional ST from consecutive slices. However, it is not specifically designed for the gene expression prediction problem that our paper focuses on. Our work is centered on predicting spatial gene expression from H&E images, which is a different type of problem as outlined here.

Overall, this study proposes mclSTExp, a multimodal deep learning method based on the Transformer and contrastive learning framework, designed to accurately predict gene expression from H&E images. Compared to existing ST frameworks, such as SPACEL [6], mclSTExp focuses more on efficiently inferring gene expression from tissue morphology. The results of our study demonstrate the method’s excellent performance in deciphering cancer-specific genes and identifying spatial domains.

**Key Points**

- We propose a multimodal deep learning approach based on Transformer and contrastive learning framework, aiming to integrate spot features, spatial location information of spots, and H&E image data for predicting spatial gene expression from H&E images.
- Utilizing the unique characteristics of ST data, particularly gene spatial location information, we treat spots as “words” and spot sequences as “sentences” containing multiple “words”. We employ a self-attention mechanism within the Transformer encoder of mclSTExp to extract spot features, and seamlessly integrate this information using learnable positional encoding.
- We infer gene expression profiles through weighted aggregation, rather than simple averaging, akin to using the softmax function.
- Our method was compared with competing approaches on multiple real ST datasets. The results demonstrate that our method achieves a 23 to 36% improvement in predicting gene expression profiles in terms of average PCC compared to the state-of-the-art methods. Additionally, our approach not only demonstrates higher accuracy in interpreting cancer-specific genes, elucidating immune-related genes, and identifying specific spatial domains, but also preserves the original gene expression patterns.

## Supplementary Material

Supplementary_Materials_Final_bbae551

## Data Availability

Three publicly available ST datasets were used in this study (Supplementary Table S1), which can be found: (i) human HER2-positive breast tumor ST data from https://github.com/almaan/her2st/. (ii) Human cutaneous squamous cell carcinoma 10x Visium data from GSE144240. (iii) 10x Genomics Visium data and Swarbrick’s Laboratory Visium data from https://doi.org/10.48610/4fb74a9.

## Code availability

All source codes used in this paper have been deposited at https://github.com/shizhiceng/mclSTExp.
