# Supplementary material for: Multimodal contrastive learning for spatial gene expression prediction using histology images
Source: Brief Bioinform. 2024 Oct 29;25(6):bbae551. doi: 10.1093/bib/bbae551 (PMC11952928; doi:10.1093/bib/bbae551)
Supplement: Supplementary_Materials_Final_bbae551 [file supplementary_materials_final_bbae551.pdf]

Supplementary Materials for

Multimodal contrastive learning for spatial gene expression prediction using histology images

Supplementary Notes

1. Key contributions of the paper

In this paper, we propose mclSTExp, a multimodal deep learning approach utilizing Transformer and contrastive learning architecture. The key contributions of the paper can be summarized as follows:

- In this study, we propose a multimodal deep learning approach based on Transformer and contrastive learning framework, aiming to integrate spot features, spatial location information of spots, and H&E image data for predicting spatial gene expression from H&E images.
- Utilizing the unique characteristics of ST data, particularly gene spatial location information, we treat spots as "words" and spot sequences as "sentences" containing multiple "words". We employ a self-attention mechanism within the Transformer encoder of mclSTExp to extract spot features, and seamlessly integrate this information using learnable positional encoding.
- We infer gene expression profiles through weighted aggregation, rather than simple averaging, akin to using the softmax function.
- Our method was compared with competing approaches on multiple real ST datasets. The results demonstrate that our method achieves a 23% to 36% improvement in predicting gene expression profiles in terms of average Pearson Correlation Coefficient (PCC) compared to the state-of-the-art methods. Additionally, our approach not only demonstrates higher accuracy in interpreting cancer-specific genes, elucidating immune-related genes, and identifying specific spatial domains, but also preserves the original gene expression patterns, thereby providing valuable insights for cancer therapy.

2. Ablation studies

To assess the contribution of each module in our proposed mclSTExp model, we conducted a detailed ablation studies on the ST dataset.

We first conducted the ablation studies on positional encodings (Table S5). We compared the performance impact of different positional encoding methods on the HER2+, cSCC, and Alex+10x datasets. The results indicate that employing learnable positional encoding methods (learnable PE) consistently yielded the best performance across all datasets. Compared to other positional encoding methods, including no encoding, sinusoidal encoding, and naive encoding, learnable PE achieved lower PCC (ACG) and PCC (HEG) scores, as well as higher MSE and MAE scores. This suggests that adopting learnable positional encoding methods better captures the spatial information of spots, thereby improving model performance in ST analysis. Particularly, on the Alex+10x dataset, using learnable PE resulted in a 15.18% improvement in ACG and a 6.65% improvement in PCC (HEG) prediction accuracy compared to not using positional encoding. This indicates that without the fusion of positional information, the model may struggle to fully utilize the positional information of spots, potentially leading to insufficient understanding of spatial structures and affecting the model's ability to model the data. In contrast, incorporating positional information fusion enables a more accurate understanding of spatial features, thereby enhancing model generalization and performance. Therefore, in ST analysis, integrating positional information fusion is essential and effective.

Furthermore, we conducted the ablation studies of the image encoder on three different datasets, namely HER2+, cSCC, and Alex+10x (Table S6). The results indicate that Denesnet121 outperforms pre-trained ViT and ResNet50 across all evaluation metrics .

Lastly, we conducted the ablation studies on distance metrics. Three different distance metrics, including L1 norm, cosine similarity, and L2 norm, were compared across the HER2+, cSCC, and Alex+10x datasets (Table S7). On the HER2+ dataset, the L2 norm method exhibited the best performance across all evaluation metrics, including PCC (ACG), PCC (HEG), MSE, and MAE, with average values of 0.2306, 0.3878, 0.6007, and 0.5868, respectively. Similarly, on the cSCC dataset and Alex+10x dataset, the L2 norm method also demonstrated superior performance, indicating its advantage in distance measurement. These findings suggest that, for these three datasets, the L2 norm, as a distance metric method, can better capture the relationships between gene expressions, thereby improving the accuracy of gene expression prediction.

We also conducted a sensitivity analysis on the top-k parameter, as illustrated in Figure S4. On both the HER2+ and cSCC datasets, mclSTExp achieved the highest PCC and the lowest MAE and MSE with $k = 200$ for all considered genes. Similarly, on the Alex+10x dataset, mclSTExp attained the highest PCC and the lowest MAE and MSE with $k = 2400$ for all considered genes.

To better explore the impact of different parameterized versions of the loss function on experimental results, we organized an ablation study on a parameterized version of the loss function. Specifically, for the loss function, we investigated the effect of the parameter λ when it is set to 0, 1, and 0.5, which is detailed in Table S8 of the supplementary materials. We found that the performance difference between $\lambda = 0$ and $\lambda = 1$ was minimal. However, using the mean of both loss functions ($\lambda = 0.5$) resulted in improved overall performance.

Supplementary Figures

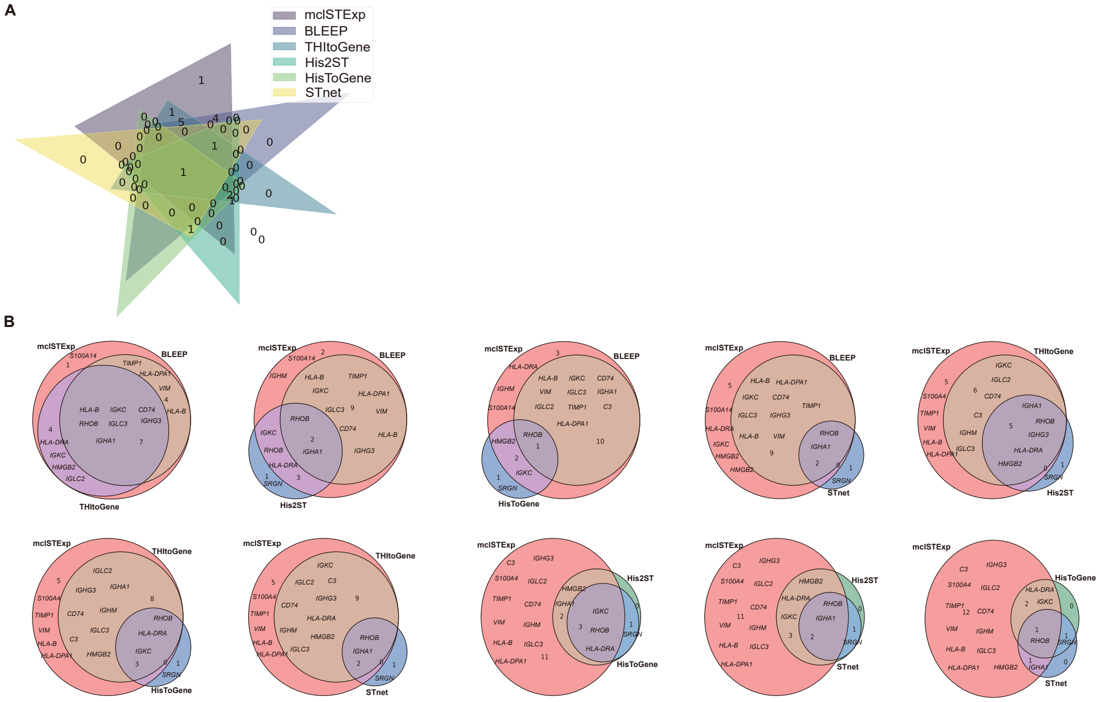

Figure S1. Venn diagrams of immune gene predictions from the HER2+ dataset, showing overlap and exclusivity across different methods. Overall comparison of immune gene predictions across six methods: mclSTExp, BLEEP, THItoGene, His2ST, HisToGene and STnet.(A) Venn diagrams of all six methods (B) Pairwise comparisons between mclSTExp and each of the other methods, showing the number of shared and unique immune genes.

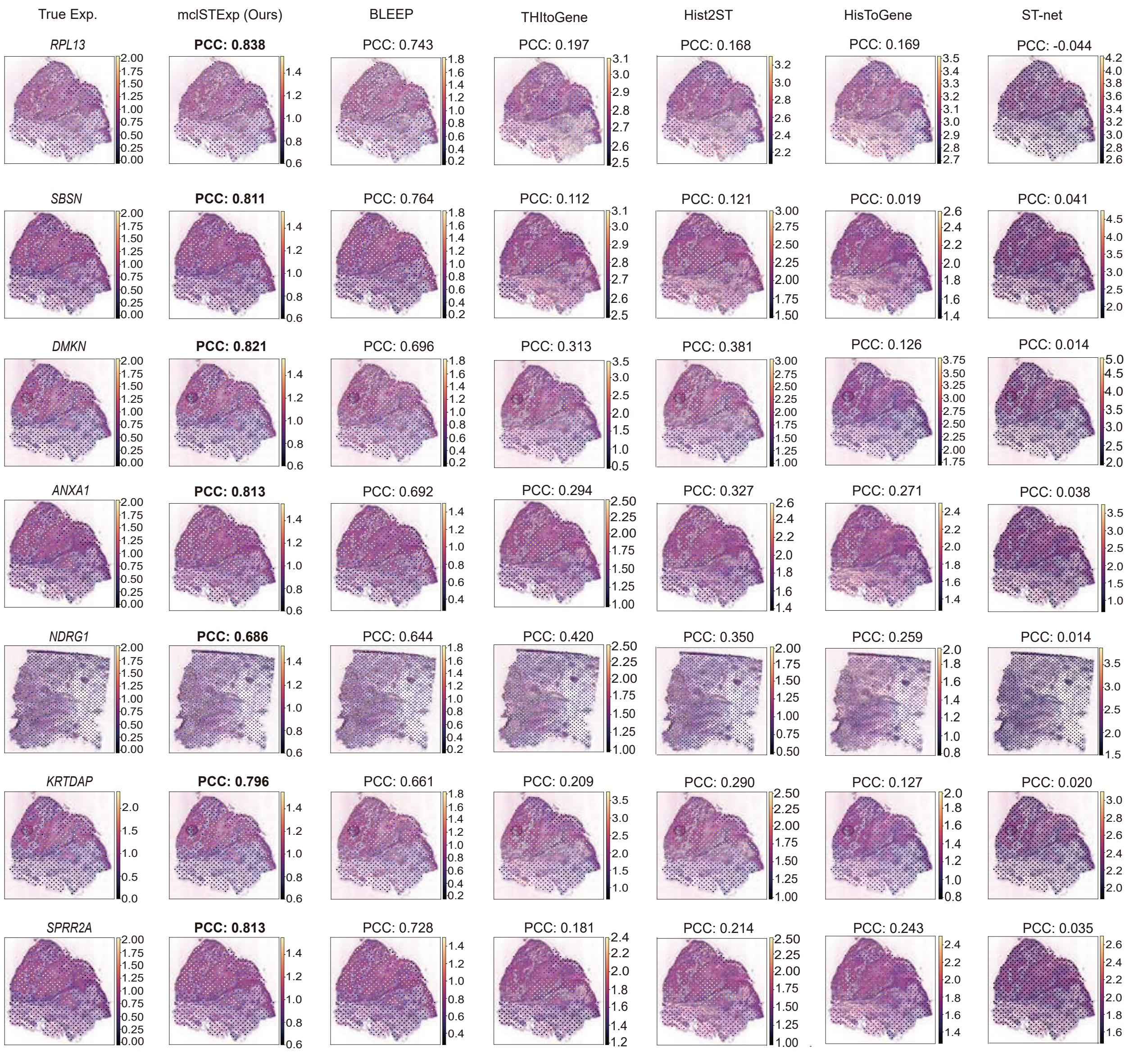

Figure S2. Visualization of the cSCC dataset by the top seven predicted genes with the highest values of average -log10 p-values across all tissue sections, where the p-value for each tissue section was obtained according to the correlation between the predicted and observed gene expression. For each of the seven genes, the tissue section that had the smallest p-value by our model was selected for visualization.

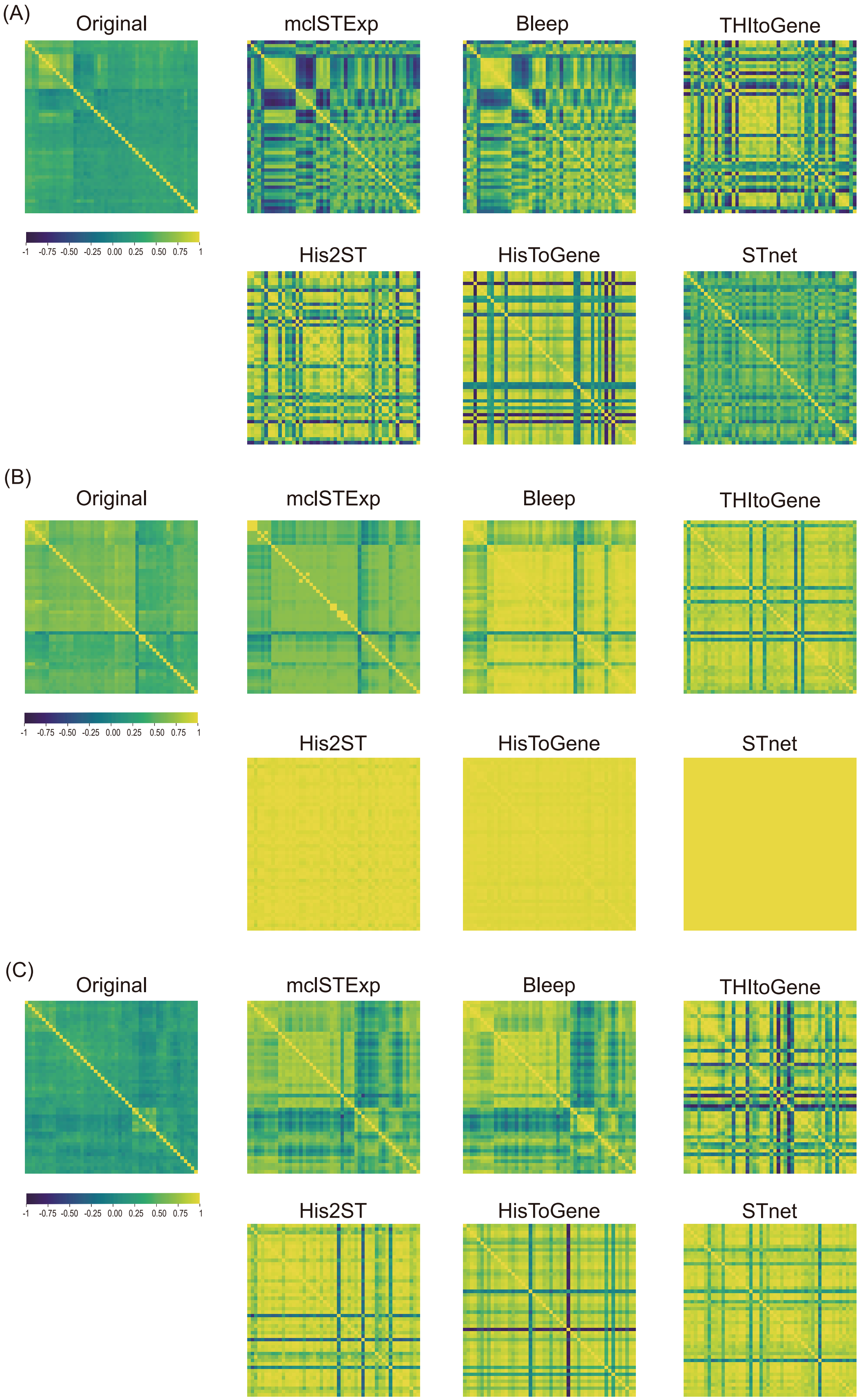

Figure S3. (A), (B), and (C) respectively represent the gene-gene correlation heatmaps calculated using the predicted expressions for the HER2+, cSCC, and Alex+10x datasets. It illustrates the effectiveness of mclSTExp in preserving gene-gene correlations, serving as evidence of its capability to maintain relevant biological heterogeneity.

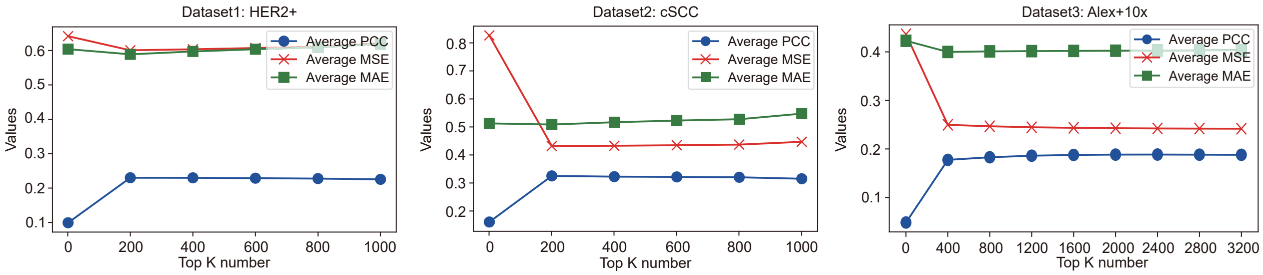

Figure S4. Ablation studies of the parameter k of mclSTExp on the HER2+, cSCC and Alex+10x datasets. The PCC, MSE, and MAE were calculated between the gene expression data predicted by mclSTExp for all considered genes (ACG) and the observed data.

Supplementary Tables

Table S1. Summary of the preprocessed datasets.

Dataset	H&E images	Resolution	Spots	Genes
HER2+ [1]	32	100 µm	11548	785
cSCC [2]	12	100 µm	8671	171
Alex+10x [3, 4]	9	55 µm	25914	685

Table S2. For all the datasets, 5-fold cross-validation was used to calculate the mean PCCs for the predicted expression levels of All Considered Genes (ACG) and the top 50 most Highly Expressed Genes (HEG), as well as the average MSE and MAE compared to the ground truth expressions.

Methods	HER2+				cSCC				Alex+10x			
	PCC(ACG)	PCC(HEG)	MSE	MAE	PCC(ACG)	PCC(HEG)	MSE	MAE	PCC(ACG)	PCC(HEG)	MSE	MAE
STnet [21]	0.0541	0.0413	0.5569	0.6486	0.0011	0.0013	0.7021	0.6584	0.0011	0.0209	0.5024	0.5235
HisToGene [15]	0.0701	0.0401	0.5359	0.6459	0.0654	0.0811	0.6978	0.6423	0.0451	0.0654	0.4856	0.5213
His2ST [22]	0.1409	0.1688	**0.5239**	0.6162	0.1652	0.1935	0.6841	0.6321	0.1015	0.1523	0.4023	0.4635
THItoGene [23]	0.1336	0.1626	0.5975	0.6313	0.1856	0.2269	0.6685	0.6149	0.0894	0.1512	0.4133	0.4821
BLEEP [24]	0.1749	0.2668	0.6154	0.6256	0.2398	0.3014	0.5321	0.5513	0.1453	0.2734	0.2689	0.4363
mclSTExp	**0.2121**	**0.3492**	0.6025	**0.6153**	**0.3113**	**0.4102**	**0.4523**	**0.5365**	**0.1745**	**0.3326**	**0.2559**	**0.4187**

Table S3. The top 50 predicted genes by mclSTExp were ranked based on the highest values of mean -log10 p-values across all tissue sections in the HER2+ dataset, where the p-value for each tissue section was obtained according to the correlation between the predicted and observed gene expression.

Rank	Gene	Average -log10 p-values	Rank	Gene	Average -log10 p-values
1	GNAS	31.7374979	26	TMBIM6	15.60088382
2	FN1	29.48300509	27	CCT4	15.48815636
3	FASN	26.5014407	28	C3	15.39573578
4	HLA-B	23.81602936	29	MUC1	15.31770172
5	SCD	23.38638176	30	MUCL1	15.30780106
6	IGKC	22.89352328	31	PRKCSH	15.29308679
7	HLA-DRA	21.18646565	32	BSG	15.21926999
8	CD74	20.83984843	33	NDUFB9	14.92890652
9	CLDN4	20.56337901	34	NDUFB2	14.89028399
10	UBA52	19.82961524	35	KRT8	14.87707216
11	HSPB1	19.37419648	36	FLNA	14.8221481
12	MYL12B	19.36653237	37	GPRC5A	14.79007249
13	STMN1	18.21896248	38	FADS2	14.76694248
14	IGLC3	17.95928677	39	LUM	14.60046262
15	IGHA1	17.82911851	40	HMGB2	14.59822067
16	IGLC2	17.82198295	41	TIMP1	14.597522
17	RHOB	17.58603295	42	AES	14.5492234
18	IGHG3	17.44433543	43	CRACR2B	14.40019307
19	VIM	17.41394217	44	POSTN	14.38532508
20	TMEM123	17.27330474	45	ITGB6	14.26458071
21	SPARC	16.66613648	46	HLA-DPA1	14.2525189
22	CLDN3	16.66542256	47	IGHM	14.20466249
23	COL3A1	16.57054216	48	ATP6AP1	14.14672836
24	CRABP2	16.19007526	49	TXNDC17	14.012606
25	NDRG1	41.05857062	50	S100A14	13.9632133

Table S4. The top 50 predicted genes by mclSTExp were ranked based on the highest values of mean -log10 p-values across all tissue sections in the cSCC dataset, where the p-value for each tissue section was obtained according to the correlation between the predicted and observed gene expression

Rank	Gene	Average -log10 p-values	Rank	Gene	Average -log10 p-values
1	RPL13	95.16443206	26	NEFL	40.50223942
2	SBSN	87.44024527	27	CASP14	40.48121358
3	DMKN	84.14861043	28	TMOD3	38.87176597
4	ANXA1	81.22854158	29	EIF5	38.8020682
5	NDRG1	79.75989866	30	MOB1A	37.78479111
6	KRTDAP	78.30615159	31	IGFL1	37.32555753
7	SPRR2A	73.20378609	32	KLF6	37.28006497
8	PI3	66.80329099	33	KIF5B	35.71358568
9	HSP90AA1	66.36587578	34	PTP4A2	35.62163211
10	ITGA6	65.53533137	35	PAICS	35.34727207
11	CALML5	61.98991283	36	STMN1	35.25029249
12	SPINK5	60.09701412	37	NAP1L1	35.12576686
13	COL1A2	54.46153265	38	PRRC2C	35.10933098
14	MSMO1	52.8260604	39	WNK1	34.9209555
15	SPRR2D	52.11175327	40	CYFIP1	34.50573809
16	ACTN4	48.61148361	41	PTHLH	34.32614153
17	HSPH1	46.36376299	42	CTNND1	34.28747832
18	ENAH	46.10806832	43	CAV1	33.40027545
19	COL3A1	45.35231055	44	RALA	31.60494295
20	FDFT1	44.67386954	45	F3	31.44476584
21	PSMA7	43.34262318	46	DIAPH1	31.32501815
22	MAFB	42.1321182	47	DDX21	31.26470445
23	EFNB1	41.69393662	48	SRP72	31.04323875
24	ZFP36L2	41.18945052	49	EIF5B	30.96488263
25	NHP2	41.05857062	50	PSMD1	30.79251656

Table S5. Ablation studies of positional encoding methods across the HER2+, cSCC, and Alex+10x datasets.

Position Encoding Methods	HER2+			
	PCC (ACG)	PCC (HEG)	MSE	MAE
W/O	0.2105 ± 0.009	0.3578 ± 0.012	0.6220 ± 0.008	0.6323 ± 0.006
Sinusiod PE [5]	0.2184 ± 0.013	0.3728 ± 0.015	0.6596 ± 0.007	0.6795 ± 0.014
Naive PE [6]	0.2262 ± 0.011	0.3796 ± 0.007	0.6162 ± 0.014	0.5975 ± 0.008
**learnable PE** [7]	**0.2322 ± 0.016**	**0.3923 ± 0.018**	**0.5815 ± 0.011**	**0.5714 ± 0.013**
Position Encoding Methods	cSCC			
	PCC (ACG)	PCC (HEG)	MSE	MAE
W/O	0.3089 ± 0.014	0.4164 ± 0.011	0.4467 ± 0.008	0.5172 ± 0.007
Sinusiod PE [5]	0.3125 ± 0.011	0.4171 ± 0.014	0.4439 ± 0.012	0.5191 ± 0.005
Naive PE [6]	0.3217 ± 0.013	0.4230 ± 0.009	0.4344 ± 0.011	0.5123 ± 0.012
**learnable PE** [7]	**0.3235 ± 0.016**	**0.4259 ± 0.010**	**0.4302 ± 0.012**	**0.5058 ± 0.014**
Position Encoding Methods	Alex+10x			
	PCC (ACG)	PCC (HEG)	MSE	MAE
W/O	0.1692 ± 0.020	0.3379 ± 0.013	0.2510 ± 0.008	0.3987 ± 0.011
Sinusiod PE [5]	0.1789 ± 0.013	0.3508 ± 0.015	0.2424 ± 0.009	0.4088 ± 0.013
Naive PE [6]	0.1795 ± 0.015	0.3496 ± 0.014	0.2398 ± 0.014	0.3961 ± 0.005
learnable PE [7]	**0.1949 ± 0.018**	**0.3604 ± 0.013**	**0.2394 ± 0.011**	**0.3897 ± 0.009**

Table S6. Ablation studies of image encoders on the HER2+, cSCC and Alex+10x datasets.

Image Encoders	HER2+			
	PCC (ACG)	PCC (HEG)	MSE	MAE
ViT	0.2236 ± 0.009	0.3750 ± 0.004	0.6007 ± 0.005	0.5853 ± 0.008
Resnet50	0.2298 ± 0.003	0.3889 ± 0.007	0.6058 ± 0.006	0.5878 ± 0.005
**Denesnet121**	**0.2312 ± 0.004**	**0.3923 ± 0.008**	**0.5821 ± 0.004**	**0.5714 ± 0.004**
Image Encoder	cSCC			
	PCC (ACG)	PCC (HEG)	MSE	MAE
ViT	0.2994 ± 0.007	0.3996 ± 0.006	0.4485 ± 0.007	0.5232 ± 0.009
Resnet50	0.3113 ± 0.005	0.4139 ± 0.005	0.4385 ± 0.004	0.5195 ± 0.008
**Denesnet121**	**0.3235 ± 0.010**	**0.4249 ± 0.009**	**0.4302 ± 0.006**	**0.5058 ± 0.005**
Image Encoder	Alex+10x			
	PCC (ACG)	PCC (HEG)	MSE	MAE
ViT	0.1745 ± 0.012	0.3023 ± 0.011	0.2724 ± 0.007	0.4454 ± 0.008
Resnet50	0.1801 ± 0.009	0.3228 ± 0.010	0.2394 ± 0.008	0.4019 ± 0.006
**Denesnet121**	**0.1948 ± 0.011**	**0.3511 ± 0.008**	**0.2373 ± 0.006**	**0.3997 ± 0.009**

Table S7. Ablation studies of distance metrics on the HER2+, cSCC and Alex+10x datasets.

Distance	HER2+			
	PCC (ACG)	PCC (HEG)	MSE	MAE
cosine	0.2301 ± 0.002	0.3871 ± 0.007	0.6009 ± 0.009	0.5889 ± 0.008
L1	0.2300 ± 0.005	0.3872 ± 0.004	0.5963 ± 0.007	0.5901 ± 0.004
**L2**	**0.2306 ± 0.004**	**0.3878 ± 0.018**	**0.5811 ± 0.006**	**0.5868 ± 0.003**
distance	cSCC			
	PCC (ACG)	PCC (HEG)	MSE	MAE
cosine	0.3262 ± 0.004	0.4124 ± 0.005	0.4317 ± 0.007	0.5063 ± 0.008
L1	0.3184 ± 0.003	0.4098 ± 0.006	0.4320 ± 0.007	0.5061 ± 0.007
**L2**	**0.3322 ± 0.007**	**0.4261 ± 0.009**	**0.4302 ± 0.005**	**0.5058 ± 0.006**
distance	Alex+10x			
	PCC (ACG)	PCC (HEG)	MSE	MAE
cosine	0.2262 ± 0.011	0.3596 ± 0.007	0.6162 ± 0.014	0.5975 ± 0.008
L1	0.2184 ± 0.013	0.3528 ± 0.015	0.6596 ± 0.007	0.6795 ± 0.014
**L2**	**0.1948 ± 0.016**	**0.3623 ± 0.018**	**0.2394 ± 0.011**	**0.3997 ± 0.013**

Table S8. Ablation studies on a parameterized version of the loss function for the HER2+, cSCC, and Alex+10x datasets.

$\lambda \times$ loss_image + (1-λ) $\times$ loss_spot	HER2+			
	PCC(ACG)	PCC(HEG)	MSE	MAE
$\lambda = 0$	0.2251 ± 0.010	0.3805 ± 0.014	0.5974 ± 0.012	0.5983 ± 0.011
$\lambda = 1$	0.2211 ± 0.011	0.3789 ± 0.013	0.6014 ± 0.011	0.5948 ± 0.015
$\lambda = 0.5$	**0.2309 ± 0.006**	**0.3891 ± 0.011**	**0.5891 ± 0.015**	**0.5864 ± 0.013**
$\lambda \times$ loss_image + (1-λ) $\times$ loss_spot	cSCC			
	PCC(ACG)	PCC(HEG)	MSE	MAE
$\lambda = 0$	0.3177 ± 0.021	0.4162 ± 0.013	0.4418 ± 0.011	0.5319 ± 0.012
$\lambda = 1$	0.3248 ± 0.017	0.4275 ± 0.014	0.4521 ± 0.010	0.5594 ± 0.014
$\lambda = 0.5$	**0.3294 ± 0.015**	**0.4281 ± 0.016**	**0.4302 ± 0.010**	**0.5208 ± 0.009**
$\lambda \times$ loss_image + (1-λ) $\times$ loss_spot	Alex+10x			
	PCC(ACG)	PCC(HEG)	MSE	MAE
$\lambda = 0$	0.1755 ± 0.007	0.3464 ± 0.015	0.2402 ± 0.010	0.3992 ± 0.013
$\lambda = 1$	0.1879 ± 0.009	0.3510 ± 0.013	0.2469 ± 0.009	0.4123 ± 0.014
$\lambda = 0.5$	**0.1948 ± 0.011**	**0.3611 ± 0.018**	**0.2329 ± 0.006**	**0.3897 ± 0.011**

References

1. Minxing Pang, Kenong Su, and Mingyao Li. Leveraging information in spatial transcriptomics to predict super-resolution gene expression from histology images in tumors. *BioRxiv*, pages 1–31, 2021.
2. Andrew L Ji, Adam J Rubin, Kim Thrane, Sizun Jiang, David L Reynolds, Robin M Meyers, Margaret G Guo, Benson M George, Annelie Mollbrink, Joseph Bergenstråhle, et al. Multimodal analysis of composition and spatial architecture in human squamous cell carcinoma. *Cell*, 182(2):497–514, 2020.
3. Amanda Janesick, Robert Shelansky, et al. High resolution mapping of the tumor microenvironment using integrated single-cell, spatial and in situ analysis. *Nature Communications*, 14(1):8353–8368, 2023.
4. Sunny Z Wu, Ghamdan Al-Eryani, Daniel Lee Roden, Simon Junankar, Kate Harvey, Alma Andersson, Aatish Thennavan, Chenfei Wang, James R Torpy, Nenad Bartonicek, et al. A single-cell and spatially resolved atlas of human breast cancers. *Nature Genetics*, 53(9):1334–1347, 2021.
5. Ashish Vaswani, Noam Shazeer, Niki Parmar, Jakob Uszkoreit, Llion Jones, Aidan N Gomez, Łukasz Kaiser, and Illia Polosukhin. Attention is all you need. In *Advances in neural information processing systems*, pages 1–15, 2017.
6. Hongzhi Wen, Wenzhuo Tang, Wei Jin, Jiayuan Ding, et al. Single cells are spatial tokens: Transformers for spatial transcriptomic data imputation. *arXiv preprint arXiv:2302.03038*, 2023.
7. Alexey Dosovitskiy, Lucas Beyer, Alexander Kolesnikov, Dirk Weissenborn, Xiaohua Zhai, Thomas Unterthiner, Mostafa Dehghani, Matthias Minderer, Georg Heigold, Sylvain Gelly, Jakob Uszkoreit, and Neil Houlsby. An image is worth 16x16 words: Transformers for image recognition at scale. In *International Conference on Learning Representations*, pages 1–22, 2021.
